# Visually Evoked Spiking Evolves While Spontaneous Ongoing Dynamics Persist

**DOI:** 10.3389/fnsys.2015.00183

**Published:** 2016-01-08

**Authors:** Raoul Huys, Viktor K. Jirsa, Ziauddin Darokhan, Sonata Valentiniene, Per E. Roland

**Affiliations:** ^1^Centre National de la Recherche Scientifique CerCo UMR 5549, Pavillon Baudot CHU PurpanToulouse, France; ^2^Faculté de Médecine, Institut de Neurosciences des Systèmes, Aix-Marseille UniversitéMarseille, France; ^3^INSERM UMR1106, Aix-Marseille UniversitéMarseille, France; ^4^Department of Neuroscience, Karolinska InstitutetSolna, Sweden; ^5^Department of Neuroscience and Pharmacology, University of CopenhagenCopenhagen, Denmark

**Keywords:** cortical states, spontaneous activity, cortical dynamics, spike trains, visual transients, neuron network stability

## Abstract

Neurons in the primary visual cortex spontaneously spike even when there are no visual stimuli. It is unknown whether the spiking evoked by visual stimuli is just a modification of the spontaneous ongoing cortical spiking dynamics or whether the spontaneous spiking state disappears and is replaced by evoked spiking. This study of laminar recordings of spontaneous spiking and visually evoked spiking of neurons in the ferret primary visual cortex shows that the spiking dynamics does not change: the spontaneous spiking as well as evoked spiking is controlled by a stable and persisting fixed point attractor. Its existence guarantees that evoked spiking return to the spontaneous state. However, the spontaneous ongoing spiking state and the visual evoked spiking states are qualitatively different and are separated by a threshold (separatrix). The functional advantage of this organization is that it avoids the need for a system reorganization following visual stimulation, and impedes the transition of spontaneous spiking to evoked spiking and the propagation of spontaneous spiking from layer 4 to layers 2–3.

## Introduction

As neurons in sensory areas of the cerebral cortex spike even in absence of input from the sensory receptors (Hubel and Wiesel, 1959; Jung, 1959), a fundamental problem is to understand how spontaneous ongoing spiking and stimulus evoked spiking relate. There are three different hypotheses on how ongoing spontaneous spiking relates to sensory evoked spiking. The first holds that rest spiking is noise upon which the spikes transmitted from sensory receptors (such as the inner ear and retina) are added. The neurons in the cortical areas then supposedly separate this noise from the spiking coming from sensory receptors (Rieke et al., 1999; Averbeck et al., 2006). The second proposes that the ongoing rest spiking is highly structured and shares properties with sensory input. Spikes coming from the retina do not drive the neuronal activity away from the spontaneous rest state, but modulate the spontaneous spiking of the cortical neurons to get an updated representation of the visual surround (Llinás and Paré, 1991; Arieli et al., 1995; Tsodyks et al., 1999; Fiser et al., 2004; Luzcak et al., 2009, 2013; Ringach, 2009; Destexhe, 2011). The third proposal is that upon visual stimulation, the rest spiking ceases and that the sensory spikes take over and drive the cortical neurons to generate a representation of the visual surround (Abeles et al., 1995; Mazor and Laurent, 2005; Jones et al., 2007; Rabinovich et al., 2009; Woodman and Jirsa, 2013). Unfortunately, experimental studies have not yet been able to select the most plausible mechanism.

Spontaneous ongoing spiking is an expression of the autonomous dynamics of the neurons in the cerebral cortex. By examining the network of neurons in the primary visual area as a dynamical system we aimed to gain novel insights into the evolution of spiking states in this network. From a dynamic point of view, we interpret the three hypotheses as follows. First, if the spontaneous ongoing spiking dynamics is qualitatively different from evoked spiking dynamics, there would be no reason to operate with the concepts of signal and noise spiking. Second if sensory evoked spiking is a modulation of spontaneous ongoing spiking, the dynamics, that is the attractor representing the spiking dynamics, may change quantitatively but not qualitatively (for example it would remain stable). Third, if the evoked spiking replaces the spontaneous ongoing spiking, the attractor dynamics should change qualitatively (for example through a bifurcation in which an unstable equilibrium replaces a stable one) upon visual stimulation. Alternatively, visual stimulation may drive the system to a different set of states without altering the spontaneous ongoing dynamics. In the later case, spiking evolves either as spontaneous state spiking or as evoked state spiking in two separate parts of state space. The spontaneous states and the evoked states then being set apart by a separatrix (i.e., a structure functioning as a threshold). To investigate these possibilities, we examined the spiking dynamics in the ferret’s visual system. Here the action potentials from the retina via the lateral geniculate nucleus interact with the spontaneous ongoing spiking of the layer 4 neurons in the primary visual area. Rather than viewing the visually evoked spiking as coding for visual attributes of the physical surround, one might see the propagation of spiking from primary visual cortex layer 4 to neurons in supragranular layers, and from here other visual areas, as spiking that ultimately drives the large network of neurons in cortical areas to an interpretation of the visual surround (Roland, 2010; Shenoy et al., 2013). In this perspective the question of whether the spontaneous ongoing spiking and the propagating evoked spiking differ dynamically becomes relevant. This question has been examined in a theoretical model of a chaotically firing neural network showing that by appropriate input, the chaotic spiking could be completely eliminated (Rajan et al., 2010). However, whereas Fdez Galán et al. (2004), Mazor and Laurent (2005) and Churchland et al. (2010) have experimentally examined spiking dynamics, we are not aware of any mathematical analysis of the stability and attractor nature of *in vivo* spontaneous ongoing spiking nor of any study showing that visually evoked spiking and spontaneous ongoing spiking is separated by a threshold (in mathematical terms, a separatrix).

## Materials and Methods

The spike data came from one group of eight adult female ferrets at rest, and exposed to one bar of 100% contrast, and to three bars of 100% contrast. An independent second group of eight other adult female ferrets, the replication group, were examined at rest, and exposed to one bar at 80% contrast.

All procedures were approved by the Stockholm Regional Ethics Committee. Adult female ferrets were anesthetized, paralyzed and craniotomized over the left visual cortical areas, as described in Harvey et al. (2009). During electrophysiological recordings, anesthesia was maintained with N_2_O:O_2_ 1:1 and 1% isofluran and body temperature, PaCO_2_, maintained as in Harvey et al. (2009) in the first group of eight ferrets. The second group of eight ferrets was maintained at 0.8% isofluran.

### Stimuli and Electrophysiology

After determining the receptive field sizes of the neurons representing the center of field of view (Harvey et al., 2009), stimuli were presented in the center of field of view on a display with a refreshment rate of 144 Hz. Stimuli were 3.7° × 3.7° white bars (64.5 cd m^−2^) lasting 250 ms on a homogenous dark-gray background (6.5 cd m^−2^) 80% contrast (second group of animals); or 64.5 cd m^−2^ on a black background, 100% contrast (first group of animals). During rest and inter-trial intervals, only the background was continuously shown. The influences of anticipation, attention and eye movements on our data were excluded.

**Figure 1:**
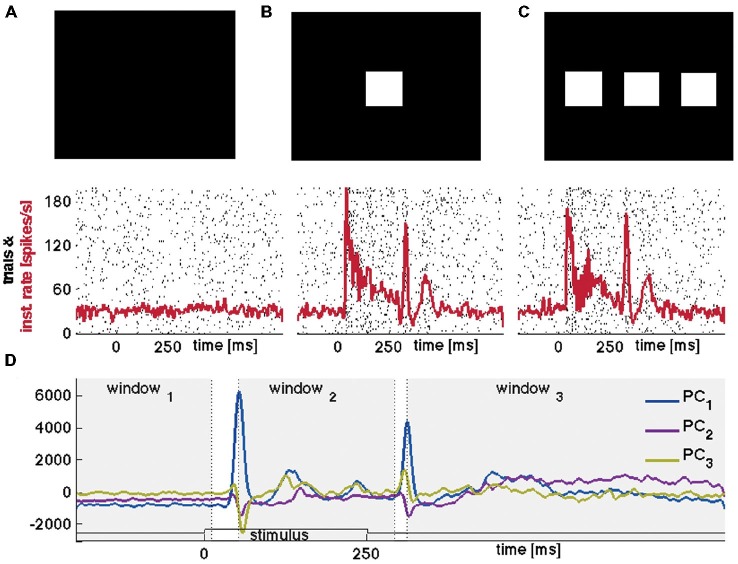
**Stimuli, raster plots, spiking rates and principal components. (A)** Raster plot of the spikes from one neuron in layer 4; 100 trials of rest (black screen), **(B)** 100 trials with 1-bar stimulus of 100% contrast, and **(C)** 100 trials with a 3-bar stimulus of 100% contrast. Animal 3: the stimuli last 250 ms. The spiking has an ON peak and an OFF peak following the stimulus on-set and off-set. The red curves show the means (across trials) of the Gaussian smoothed firing rate histograms (see “Materials and Methods” Section). **(D)** Time evolutions of principal components 1–3 for all trials for the 1-bar condition. Animal 4: the stimulus appears at time 0. The gray figure-background define three time intervals [−200 to 10 ms], [ON-peak to 270 ms], [OFF-peak to 800 ms] used in the vector field analysis (Figure 6).

The experiments in the first group had three conditions: (1) a black background; (2) one bar presented in the center of field of view at 100% contrast; and (3) three bars presented with 100% contrast and center to center distance of 3.7° along the horizontal meridian in the field of view with the center bar at the center of field of view. The two different stimuli had the purpose of replicating the spiking dynamics. The 3-bar stimulus differed from the 1-bar stimulus only by having three identical bars (Figure 1). Both stimuli were appearing abruptly and therefore expected to produce clear visual transients. The second group had two conditions: (1) a dark-gray background; and (2) one bar presented in the center of field of view at 80% contrast. The presentations of conditions were random, but balanced such that each condition was presented 100 times (50 times, replication group) with a 10 s interval between trials.

We recorded from 125 multi-units with a single shank 16 channel laminar electrode (2–3 MΩ) perpendicular to the cortical surface of areas 17 and 18 where neurons had receptive fields <1.5° and were reacting to small bars in the center of field of view. We recorded 85 multi-units in the replication group. Recording sites were separated by 100 μm and current source densities were obtained as described in Harvey et al. (2009) to select five recording channels representing the input layer (layer 4 and lower layer 3) and with a 200 μm gap three leads representing layers 2–3. The location of the laminar position of the leads was indirect and their laminar location consequently an estimate.

### Data Analysis

The spikes of the first group were spike-sorted off-line (Quiroga et al., 2004). Refractory periods >3 ms. For each animal, penetration, condition, and trial, the spike time histograms (in 1 ms bins) were smoothed using a moving average with Gaussian kernel, window 15 ms; *σ* = 6 ms.

#### Principal Component Analysis (PCA)

Each PCA was performed as follows: for a given *N*-dimensional state vector, we computed the covariance matrix (which includes subtracting the mean value of each time series), normalized it (by dividing it through the sum of its diagonal), and next computed the covariance matrix’s *N* eigenvalues *λ* and (*N* × *N*) eigenvectors *v*. The corresponding time evolutions (or projections) were obtained by multiplying the data (state vector) and the eigenvectors (Daffertshofer et al., 2004). Each principal component, PC, accounts for a proportion of the total variance in the data, captured by the (associated) eigenvalues *λ*.

**Figure 2:**
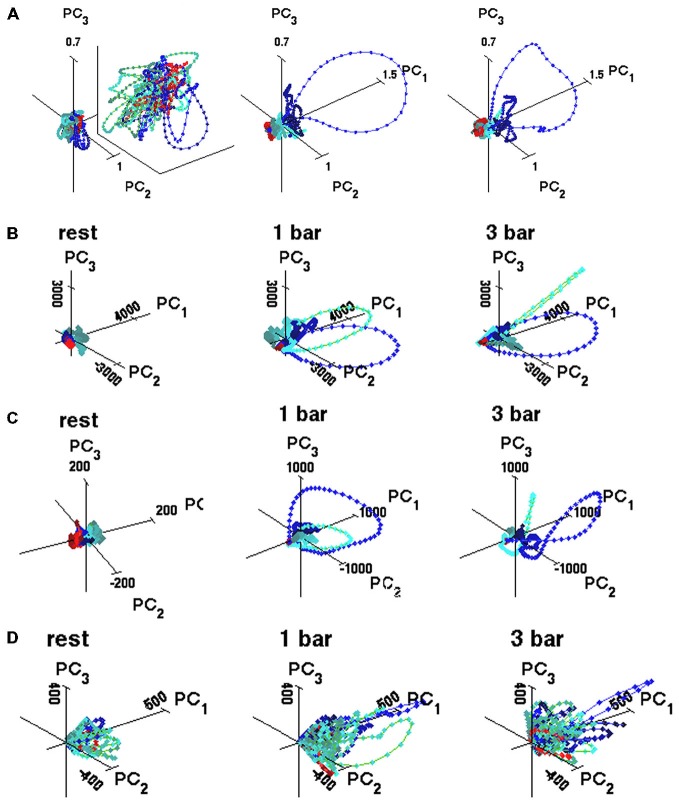
**State space trajectories. (A)** Representative trajectories based on five simultaneously recorded multi-units, enlarged for the rest condition (multi-unit PCA see “Materials and Methods” Section). Trajectories made of connected points with 1 ms spacing. Red, blue, and green correspond to windows 1, 2 and 3, respectively, identified in Figure 1D. The state space is made of the orthogonal axes of the first three principal components. Animal 8: the first three components accounted for 18% (rest), 80% (1-bar), and 82% (3-bar) of the total variance. **(B)** Spiking trajectories calculated across 100 trials for one neuron. Animal 1 **(C)** Trajectories of all multi-units in the experimental group (125 multi-units). **(D)** Trajectories of seven simultaneously recorded neurons from layer 4 (7-dimensional) for single trial (PCA see “Materials and Methods” Section). The first three components accounted for 67% (rest), 70% (1-bar), and 81% (3-bar) of the total variance. Principal component units on the axes. Color coding of trajectories: red from −200 to 10 ms after stimulus onset; blue, from ON-peak to 270 ms; green from OFF peak to 800 ms.

We performed three types of PCA. with the purpose of creating different state spaces and examining the robustness of our results. (1) In the *multi-unit* PCA, the simultaneously recorded multi-units of the five leads in layer 4 were organized in a 1000 × 500 state vector [i.e., 1000 ms × (five leads × 100 trials) for one condition]. For Figure 2A, the trajectory of the first three PCs are plotted in a 3-dimensional state space, separately for each condition. For the state space of the trajectory tangent vectors (see below) the data matrix was 1000 × 1500 for the plotting of the rest, 1-bar and 3-bar condition. The first two components accounted for 60–76% of the total variance. (2) For the *average multi-un*it PCA, the spiking time series of all 100 trials was averaged for each lead prior to the subtraction of the mean. This gave a 1000 × 125 state vector [i.e., 1000 ms × (all leads from all animals) for one condition]. After PCA, the first three projections were plotted in 3D space (Figure 2). (3) In the *trial × multiunit* PCA, the analysis was done per animal for the electrode penetrations in area 17/18. If the animal had two penetrations, it had a total of 200 trials. Each trial results in five time series, one for each lead. The state vector then thus comprises 1000 time series. The first two or three principal components (for a given animal) then were plotted in 2D or 3D state space, respectively. This *trial × multiunit* PCA was used for the vector field analysis (see below).

The PCA created state spaces with orthogonal axes in which we observed the time evolving spiking trajectories. The PCA transformation of the spiking rates implies that, although there is a correlation between the original instantaneous rates and the spread of a principal component, there is no unambiguous one-to-one mapping between the instantaneous spiking rate *r(t)* and the vectors and trajectories in PC state space. Consequently, statements based on *r(t)*, or its mean value, cannot predict the spiking dynamics.

#### Spike Rate Vector Fields

The vector field analysis was performed on reconstructed data (projections) from the *trial × multiunit* PCA (thus, for each animal and condition separately). For the eigenvectors $v_{i}^{k}$, where *i* = 1:5 indexes the five leads, and *k* 2 (i.e., PC 1 and 2), novel 2-dimensional data *q*_1, 2_*(t)* were constructed by projecting each trial’s five-dimensional data vector *x_i_* = 1…5 *(t)* (lead 1–5) onto the eigenvectors $v_{i}^{k}$.

The computation of the vector fields is based on the Kramers-Moyal expansion (Daffertshofer, 2010), and includes computing *P*(x, y, t ∣ *x*_0_, *y*_0_, *t*_0_), that is, the conditional probability that the system is at state (*x*, *y*) at a time *t* given its state (*x*_0_, *y*_0_) an earlier time step *t*_0_. In our case, *x* and *y* equalled *q*_1_(*t*) and *q*_2_(*t*). For each trial, we computed the conditional probabilities *P* for the 441 points of a 21 by 21 grid, after which the average conditional probabilities were calculated across trials (for each condition and animal). The so-called drift coefficients (representing the system’s deterministic dynamics) were computed according to:

$$D_{x}(x,y)\;=\;\underset{\tau \to 0}{\lim}\frac{1}{\tau }\int \int (x^{\prime }-x)P\left(x^{\prime },y^{\prime },t+\tau |x,y,t\right)dx^{\prime }dy^{\prime }$$

$$D_{y}(x,y)\;=\;\underset{\tau \to 0}{\lim}\frac{1}{\tau }\int \int (y^{\prime }-y)P\left(x^{\prime },y^{\prime },t+\tau |x,y,t\right)dx^{\prime }dy^{\prime }$$

The coefficients *D*_x_ and *D*_y_ provide the numerical representations of the system’s 2-dimensional vector field (Figure 6).

#### Estimation of the Position of the Separatrix

**Figure 3:**
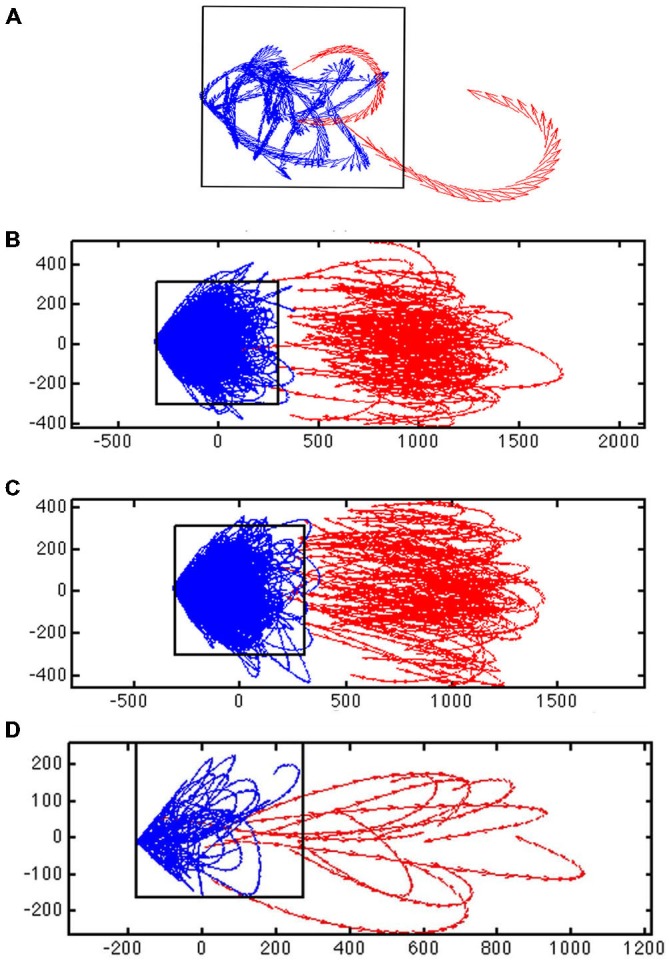
**Evidence for a separatrix. (A)** Trajectory tangent vectors (see “Materials and Methods” Section) of two single trials from 30–50 ms after stimulation with 1-bar (red), and in pre-stimulus time (blue), in a 2-dimensional state space formed by the first and second principal component (see “Materials and Methods” Section). The directions of the vectors are marked with arrows for each ms. In one stimulus trial the vectors bend off and stay within the attraction of the fixed point (and point towards the fixed point); in the other trial spiking becomes evoked (producing a diverging vector flow away from the fixed point). The box shows the estimated position of the separatrix (based on 100 trials of rest; animal 7). **(B)** Trajectory tangent vectors of 100 trials 30–50 ms after one bar stimulus onset (red). The red vectors point away from the blue vectors and the fixed point. Different metric of the distances to fixed point on the *x*- and *y*-axes. Animal 4 **(C)** Idem for the 3-bar condition. Animal 4 **(D)** Close up of the trajectory tangent vectors from 10 trials all of which are evoked. The red tangent vectors cross the separatrix from various positions inside the separatrix on their way to escape the spontaneous state and return by another path. Note the divergent vector flows at the position of the separatrix.

To examine whether the PC state space of each simultaneously recorded multi-unit data set had a separatrix with diverging vectors of velocity on either side, we calculated the trajectory tangent velocity vectors. The PCs were calculated as multi-unit PCA across the three conditions rest, 1-bar and 3-bar stimulation (see above). For each single trial, the trajectory tangent vectors were calculated as the temporal derivative at successive positions of the trajectory in 1 ms steps. The length of the resultant vector thus was the instantaneous velocity as shown as one arrow per ms in Figure 3. These trajectory tangent velocity vectors are different from the vectors used to form the vector fields. As PC1 and PC2 together accounted for more than 60% of the variance, we plotted the velocity vectors for the 100 trials of each data set of five multi-units in 2D. As the progression of the rest state velocity vectors was slow, the arrows became very dense in Figure 3.

For a given time interval, e.g., from 30–50 ms after the start of the stimulus, the trajectory vectors in two dimensions in many single trials in the stimulus conditions point away from the fixed-point. Conversely, the vectors in the rest and pre-stimulus interval when they reached a certain distance from (0, 0) pointed towards (0, 0).

**Figure 4:**
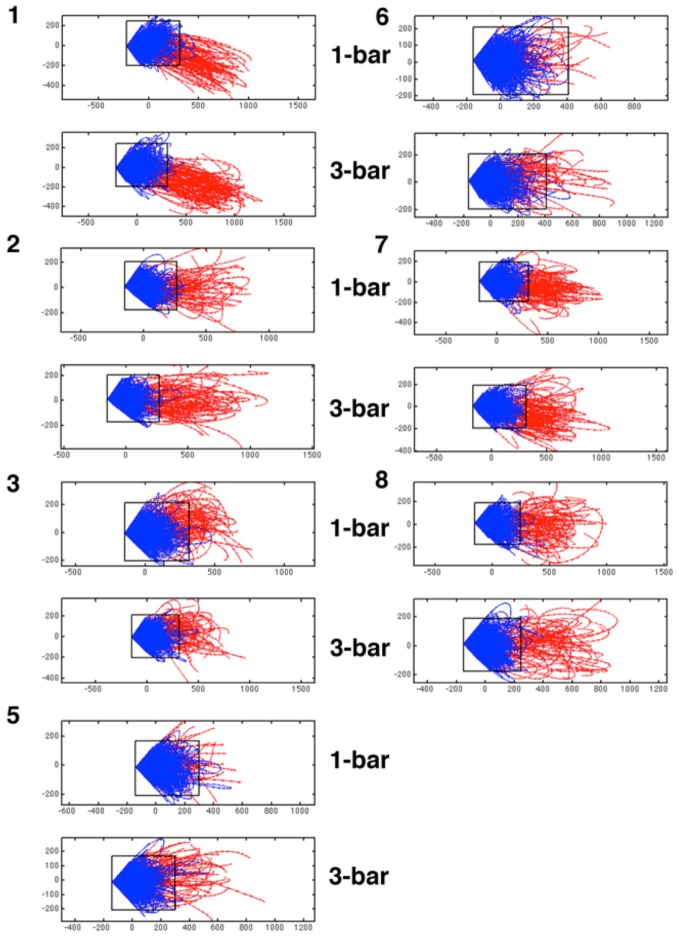
**Diverging trajectory velocity vectors.** Examples of trajectory tangent velocity vectors in 2D showing separatrices for datasets of five leads (5 multi-units) in layer 4 for the remaining animals in the experimental group. Trajectory velocity vectors colored red in the interval 30–50 ms after stimulus onset. The rectangle delimiting the spontaneous ongoing tangent vectors from the evoked are also shown in the 1-bar and 3-bar plots. The data sets are numbered with animal number. As the state space was calculated for each data set of simultaneously recorded multi-units separately, the state spaces have different metrics and cannot be transformed to a single space.

The separatrix is located between the space occupied by the spontaneous ongoing spiking trajectory vectors and the space occupied by the evoked trials’ trajectory vectors outside the separatrix. We adjusted a rectangular box to a false discovery rate of 0.05 (Benjamini and Hochberg, 1995) for the vertical sides and 0.05 for the horizontal sides of the box of rest trials being outside the box (once in the 1000 ms of sampling). This gave a total false discovery rate of approximately 0.1 for the estimate of the position of the separatrix (Figures 3, 4).

#### Peak Firing Rate Frequency Distributions Following Stimulus Onset

The peak firing rates were extracted from the original but smoothed time series. Smoothing was achieved via a sample-by-sample moving average with a window size of 15 samples (using Matlab^®^’s “gausswin” function with alpha = 2). For each animal and condition, we extracted the maximum rate of the ON peak (which occurred just following stimulus onset) in each lead of each stimulus trial, and calculated the corresponding frequency distributions (bins size: 15 spikes/s) of all trials. The rest trial peak rate was determined as the maximum in an interval from −10 to +10 ms of the (average) time for the ON peaks (as observed in the stimulus trials). We verified that it did not matter at which point the interval was chosen for the no-stimulus trials. The frequency distributions were then averaged across penetrations and single units per animal. The comparisons of the 100 and 80% contrast conditions (Figure 8) were made on the smoothed times series of the multiunit data.

For the smoothing of the raw spike data, Matlab^®^ alpha values from 1.5–3.5 were tried, but these did not qualitatively alter the peak rate distributions relative to alpha = 2. In addition, we also computed the frequency distributions for bin size 10, 20, 30, 40, and 50 spikes/s, which did not qualitatively affect the frequency distributions either.

#### A Topological Equivalent Model and Simulations

Topological equivalence is a concept that mathematically defines qualitative similarity in dynamical systems (Guckenheimer and Holmes, 1983). We simulated two planar dynamical systems; one comprises a linear flow with a stable fixed point; the other comprises a nonlinear flow with a stable fixed point and a separatrix at one side. The latter systems are known as excitable systems in nonlinear dynamic systems theory and are often used as a phenomenological representation of neural functioning for single neurons (Fitz-Hugh, 1961; Nagumo et al., 1962) but also for population dynamics (Curto et al., 2009). Here, we interpret the model in the latter sense. A mathematical representation of such phase planar systems reads:

$$\dot{x}\;=\;\tau (y+bx-cx^{3})$$

$$\dot{y}\;=\;-(x-a-I)/\tau$$

where *a, b* and *c* are parameters that determine the system’s topology. In terms of a network’s dynamics, *x* may be interpreted as the activity of a neuronal population and *y* as a recovery variable (related to ion channel mechanisms), and *τ* is a time scale parameter. The system with the nonlinear phase flow is given by *a* = 1.05, *b* = 1, *c =* 1/3, *τ =* 3 generating a stable fixed point with a separatrix in its neighborhood. Under these constraints, the system settles at the fixed point and will only traverse a trajectory through phase space if brought across the separatrix by the input *I* (or noise). When the system crosses the separatrix and the “evoked state” is activated, then a fast large-amplitude excursion occurs, followed by a slow return towards the fixed point. Together, separatrix, fast deviation and slow return after large-amplitude excursion, these elements are the key characteristics of excitable systems. The system with a linear fixed point is given for *a* = 1.05, *b* = −1, *c* = 0, *τ* = 1.

Equation 1 was simulated 500 times for both parameter settings with different stimulation strength *I* = 0–5 (step size 0.5) and varying Gaussian noise strength (Q, 0.0025, 0.005, 0.01, 0.02, 0.03, 0.04, and 0.05). Model simulations ran from *t* = 0 to *t* = 100, with a fixed 0.05 time step (this was assured by using a time vector rather specifying the integration first and last point only). The stimulation of *I* consisted of a block pulse (duration 0.5; delivered at *t* = 20). The simulations were performed using Matlab’s “ode15s” integrator (a small adaption allowed us to additively include noise at each integration step to the 2nd state variable, i.e., *y*, using the Euler-Maruyama algorithm). In both scenarios (linear and nonlinear), the system traverses a trajectory through phase space following the stimulation. This is always so in the linear case and most often, but not always so in the nonlinear case due to the separatrix. We extracted the maximal amplitude of the *x* variable (following the stimulation) and made a frequency distribution (histogram; bins width 0.1) of these maxima (for each noise level and “stimulation strength level” separately). The noise level used in the distributions plotted in this figure is *Q* = 0.0025.

Importantly, in the fixed point regime the entire phase space contains a stable fixed point and a separatrix (Figure 9). In this regime, the model predicts that without stimulation a just few trajectories will cross the separatrix, and conversely, stimulation notwithstanding a just few trajectories will not cross the separatrix. Qualitatively, these predictions are robust under moderate variations of parameters *τ* and *b* as long as *τ* > 1. Variation in these parameters does to some extent alter the likelihood of the system to cross the separatrix, and thus the ratio between the height of the peaks in the distribution in Figure 9B (left panel). Preservation of the qualitative results for the linear model also holds for these parameter variations. Variation in parameter *a*, which should be larger than one to guarantee the fixed point’s stability, determines the position of the fixed point relative to the separatrix: increasing *a* increases the fixed point—separatrix distance. The larger the distance, the less likely it is that a given stimulation gets the system across the separatrix. Furthermore, stronger stimulation should increase the chance of crossing the separatrix but barely (if at all) change the peak-firing rate. These predictions are consistent with our data (Figure 8). The simulated peak firing rate distributions show two peaks but the 0 Hz peak is not present, which is a shortcoming of the model. Regardless, one key aspect of the model (within a bounded region of its parameter space), i.e., the stable fixed point and separatrix, and thus persistence of the rest attractor in the evoked state in state space allow for a novel conceptualization of the issue how the rest state and the evoked state are linked to each other.

## Results

The eight adult ferrets were either at rest, i.e., exposed to a black screen, or exposed to suddenly appearing visual stimuli at 100% contrast. At rest there was no visual stimulation hence any ongoing spiking is by definition spontaneous. The 250 ms lasting 1-bar and 3-bar stimuli were expected to evoke similar transient spiking dynamics because they were both high contrast and abruptly starting stimuli. Figures 1A–C shows raster diagrams for 100 trials in rest, 100 trials after stimulation with one bar and 100 trials after stimulation with three bars of typical neurons in the middle layer of area 17.

### Spontaneous Ongoing Spiking Trajectories and Visually Evoked Spiking Trajectories Evolve in Different Parts of State-Space

To test whether the spontaneous ongoing spiking and the visually evoked spiking are dynamically separate, we examined the evolution of the spiking in state-space. The spiking of all simultaneously recorded neurons is mathematically represented in a multi-dimensional state space. Each point in state space corresponds to one state of the network of neurons at the area 17/18-border, and conversely; to each state of the network there is one point that is element in state space. The granular layer in the ferret area 17 extends some 500–600 μm (Innocenti et al., 2002) corresponding roughly to five leads of the electrode. For each electrode penetration, five multi-units in the granular layer were simultaneously recorded. To visualize their corresponding spiking evolution we performed a PCA (Figure 1D) of the five time series (see “Materials and Methods” Section) and plotted the evolution of the first three components represented as a trajectory in 3-dimensional space (Figure 2A).

At rest and prior to visual stimulation, the trajectory evolved erratically around (0, 0, 0). This spontaneous spiking remained confined to a small part of the state space (Figures 2A–D). In both stimulus conditions, ~30 ms after stimulus onset, the 3D spiking trajectories quickly escape the zone of the ongoing spiking and traverse large parts of the state space outside the spontaneous spiking zone. The spiking trajectories slow down and return along another route to the state-space near (0, 0, 0) resuming the spontaneous behavior (Figures 2A–D). Approximately 60 ms after the stimulus offset, the spiking trajectory again escapes the spontaneous spiking state space and traverses a different part of the state space, for thereafter to return (Figures 2A–D). These qualitative differences between the spontaneous and the evoked trajectories were reproduced in all sets of simultaneously recorded neurons. When the PCA was done to reveal the evolution of spiking in single trials (Figure 2D, see “Materials and Methods” Section), or in single neurons (Figure 2B), or in all multi-units (Figure 2C), the trajectories always maintained these differences between the spontaneous and evoked spiking. Thus, the spontaneous spiking trajectories (i.e., at rest and in the pre-stimulus interval) typically evolved erratically around (0, 0, 0) whereas the visually evoked trajectories evolved fast and explored large parts of state-space and subsequently returned more slowly towards (0, 0, 0). This behavior was present in single trials, in single neurons, and in the collective spiking dynamics of 5–8 simultaneously recorded neurons and in five simultaneously recorded multi-units. The evoked spiking started some 25–35 ms after the start of the stimuli.

### A Threshold (Separatrix) Separates Spontaneous Ongoing Spiking from Evoked Spiking

In the 1-bar and 3-bar conditions, the spiking explored a larger domain in state space (Figure 3). This means that the stimuli drive the spiking away from the state space of spontaneous ongoing spiking and away from (0, 0, 0). At rest and during the pre-stimulus time the dynamics evolved around (0, 0, 0). To test the hypothesis that the spontaneous type and transient evoked type of trajectories evolved in separate domains of the state space, we calculated the velocity vectors of the spiking trajectories of the simultaneously recorded five multi-units (see “Materials and Methods” Section). Mathematically, the state space may be divided into separate domains by “borders” (separatrices) with diverging phase flows on each side. A separatrix acts as a threshold impeding the motion from one (confined) part of the state space towards another.

If there is a separatrix, the trajectory tangent vectors (methods) will typically bend off when they approach it and point within the space defined by the separatrix. Under the influence of perturbations, such as stimulation or through fluctuations, the trajectory tangent vectors typically cross the separatrix and enter the state space adjacent to it (Figure 3A). From 30–50 ms after stimulus onset, in the majority of the trials, the tangent vectors departed and pointed away from the zone occupied by the rest and pre-stimulus spontaneous type trajectories (Figures 3B,C). This happened in 1916 trials out of 2500 in the 1-bar condition, in 2021 trials of a total of 2500 trials in the 3-bar condition and in 277 trials in the rest condition. Conversely, in the rest and pre-stimulus time the spontaneous tangent vectors when they reached a certain distance from (0, 0) bend off pointed inside and towards (0, 0). This happened for 2223 trials out of 2500 spontaneous ongoing spiking trials and for 584 trials in the 1-bar condition and 479 trials in the 3-bar condition. Starting 20 ms after stimulus onset, the different trial vectors, at this point in time, were at different positions inside the “spontaneous domain” (red vectors in Figure 3D). The position inside this domain did not seem to matter, because trajectories could transit to the “evoked domain” from various positions (Figures 3D, 4). The separation of the two state space domains, i.e., the position of the separatrix, varied somewhat among the data sets depending on the spread of the data. The mean area of the rectangular area bordered by the separatrix was 1.9 × 10^5^ with standard deviation 0.5 × 10^5^.

The diverging tangent vectors were present in all sets of layer 4 multi-units recorded simultaneously (Figure 4) and in the layer 4 multi-units recoded in the eight other ferrets (the replication group; data not shown). These results are indicative of a separatrix between the zone of state space occupied by the spontaneous ongoing spiking and the state-space occupied by the visual evoked spiking. Therefore the rest and stimulus evoked spiking evolve in two different domains of state space. The two domains are adjacent, but separated by the separatrix. The approximate position of the separatrix is shown in Figures 3, 4. Thus, the spontaneous ongoing spiking states and the visually evoked states evolve in two different domains of state space separated by a separatrix.

**Figure 5:**
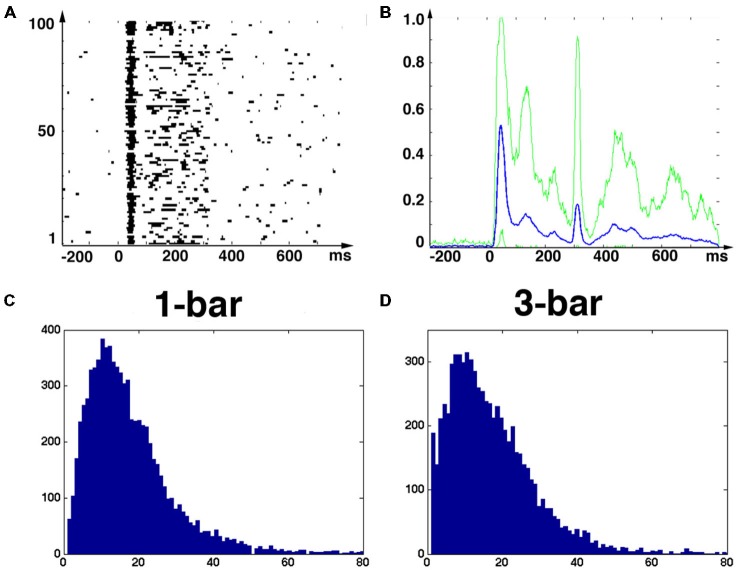
**Time the spiking trajectories were in the evoked state. (A)** Raster plot of time the spiking trajectories spent evoked in each of the 100 trials. One trial can enter the evoked states several times. Animal 8 **(B)** Proportion of trials being evoked −200 to 800 ms after start of stimulus (1-bar). All multi-unit datasets. Mean (blue) and square root of the variance (green). **(C)** Time spent evoked from 20 to 800 ms after stimulus onset for all evoked single trials; 1-bar stimulus **(D)** idem for 3-bar stimulus. *Y*-axis number of trials, *X*-axis duration of time spent evoked in ms.

We then separated spiking trials, irrespective of the experimental conditions, into two categories, spontaneous ongoing trials and evoked trials, depending on whether they did not or did cross the separatrix in the post-stimulus period (see “Materials and Methods” Section). The number of trials being in the evoked state varied somewhat over time. In single trials the system evolved in the evoked state for limited durations. However most trials evolved in the evoked state corresponding in time to the ON transient (Figures 5A,B).

### The Spontaneous Ongoing Spiking Evolves Around a Stable Fixed Point that Persists During the Evoked States

**Figure 6:**
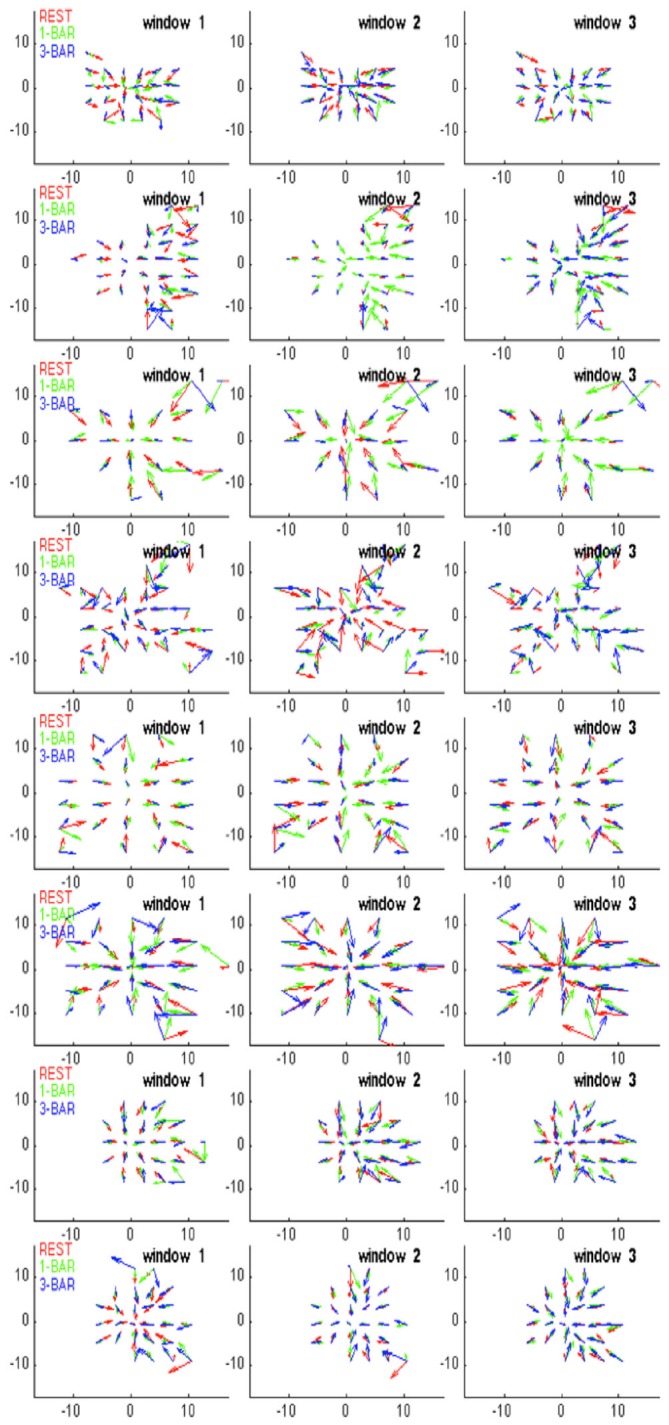
**Vector fields showing a stable fixed-point (see “Materials and Methods” Section).** Vector fields in 2-dimensions spanned by PC1 and PC2 for the rest and 100% contrast stimuli conditions in the three time windows in Figure 1D. These windows correspond to the pre-stimulus time, and the time periods after the peak of the ON and OFF response, respectively. Only the state space restricted to the fixed point and its immediate surround is shown, Red, green, and blue vectors represent the rest (black screen), 1-bar, and 3-bar condition, respectively. For each state space location, vectors are plotted only if present for all three conditions. The criterion was that all conditions i.e., rest, 1-bar, and 3-bar, must have sufficient statistics to calculate a resultant vector direction for each grid point. The vector fields are a graphical representation of the system’s deterministic) dynamics, and indicate the system(s) transition as a function of its position in state space. In all conditions and in each time window the vectors converge towards to (0, 0), indicating the existence of a stable fixed point at (0, 0). The vector field was calculated for all trials for all single neurons per animal. The vector fields show the consistent network dynamics. Animals from the top: 1–8.

The spontaneous ongoing spiking trajectories during rest and prior to the stimulus only made small excursions from (0, 0, 0), which suggests that (0, 0, 0) is a stable fixed point, i.e., an attractor. When the distance to (0, 0, 0) increases, the attraction brings the spiking immediately back near the fixed point (Figures 2–4). To test the hypothesis that the spontaneous ongoing spiking dynamics quantitatively evolves around a stable fixed-point, we calculated the vector fields in 2-dimensional state space for the time intervals specified in Figure 1D for all trials (see “Materials and Methods” Section). These vector fields are shown in Figure 6. Within the spontaneous ongoing spiking domain, the vectors converged towards one single point (0, 0) in all three conditions (rest, 1-bar, 3-bar) and in all (three) time windows of (Figures 1D, 6).

This shows mathematically that (0, 0) is a stable fixed point in the three intervals of Figure 1D. This suggests that the rest state attractor persists even in those trials in which the spiking initially escaped the attraction of the fixed point crossed the separatrix and thereby became evoked. As seen from Figures 3, 4, the trajectory velocity vectors did not converge towards other fixed points in the state space. This is also evident from the visually evoked spiking trajectories (Figure 2). This implies that the stable fixed point at (0, 0) was the only attractor in state space.

When the spiking dynamics escapes the fixed-point attraction, it might be because the fixed point in these trials gets unstable or dissolves. If this happens, the spiking will not return to the rest zone, because there would be no attraction to bring the spiking back. This possibility, however, is disproved by our results: first, the spontaneous spiking attractor persisted in the evoked state and continued to attract spiking in the evoked state as evident from the vector field analysis of all animals (Figure 6). Second, when outside the separatrix, the tangent velocity vectors sooner or later bended and then pointed towards the fixed-point, meaning that the system spent shorter or longer time in the evoked state, but was always under the fixed-point attraction (Figures 2–4). Third, for the trials that did escape the spontaneous ongoing state’s basin of attraction and entered the visually evoked state, we calculated the time the trials spent in the evoked state, i.e., from the time the trajectory crossed the separatrix on the way out until the same trajectory crossed the separatrix on its way in. These times spent evoked varied up to over 80 ms (Figures 5C,D). As soon as the spiking in single neurons, or the multi-unit activity, stays beyond the separatrix, the spiking transforms into the spontaneous type dynamics (Figure 2). Thus, our results are not compatible with the hypotheses that the rest state attractor disappears or gets unstable for a few ms when the spiking enters the visually evoked state. Furthermore, these hypotheses would imply that trajectories would (exponentially) diverge from the (0, 0, 0) point in all stimulus trials and in no single trial in the rest condition, which was not the case.

In the region demarcated by the separatrix (on one side of the fixed point), the vector-field vectors point towards the fixed point (Figure 6). Occasionally there will be trials in which the spontaneous ongoing spiking trajectories cross the separatrix. In these cases the time spent as evoked is short (Figure 5). Similarly there are trials for which the visual transients failed to drive the trajectories and hence the tangent velocity vectors beyond the separatrix, and stimulus trials in which the time spent as evoked was equally short (Figures 3A, 4). For most stimulus trials though, the escape from spontaneous ongoing spiking state domain was fast <10 ms (Figures 3–5).

### Putative Effects of the Spiking Dynamics on Peak Spiking Rates

**Figure 7:**
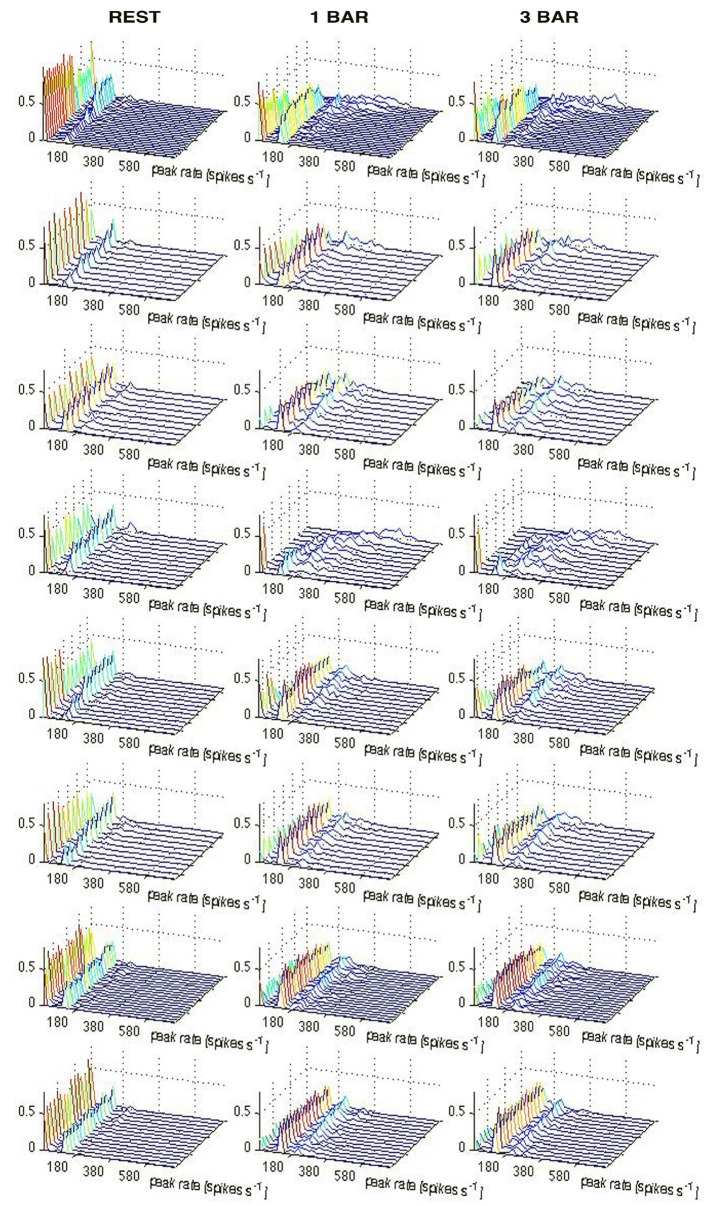
**Distributions of ON peak firing rates for all neurons.** One row per animal. The single neurons are sorted according to peak rates (*x*-axis); *y*-axis, individual neurons; *z*-axis, proportion of trials. Due to the Gaussian filter used to create the instantaneous rates (see “Materials and Methods” Section), trials with no spikes occupies from 0–10 on the *x*-axis. Note the uniformity of the peak rate distributions at rest with a large proportion of trials with no spikes and a maximum of ~120 spikes s^−1^. The proportion of trials with no spikes diminishes in all neurons under the stimulus conditions and the proportion of trials with peak rates >140 spikes s^−1^ increase in all animals. Animals from the top: 1–8.

So far our results have shown that the layer 4 network of neurons in areas 17/18 mathematically behaves as a mono-stable dynamical system (one permanent fixed-point) with a separatrix separating the spontaneous ongoing spiking states from the visually evoked spiking states. If a separatrix indeed exists, and is typically but not invariantly crossed under stimulation but not otherwise, then the peak firing rates should show a bi-modal distribution. For each of the single neurons we calculated the peak rate for each of the 100 trials for each condition. The peak rates were detected in a 20 ms time interval after the start of stimulation when the rates were maximal (the peak-spike rate time point ± 10 ms, see “Materials and Methods” Section). Figure 7 shows the peak rate distributions for these 20 ms windows. First, all neurons had trials in which they clearly responded to the stimuli. Second, all neurons sampled had trials during which they fired during rest and trials in which they were silent (Figure 7). Consequently, the spiking during rest defines the spontaneous ongoing spiking attraction, as also evident from Figures 3–5. Therefore, the rest attractor is something different from the neurons being silent (i.e., not spiking).

**Figure 8:**
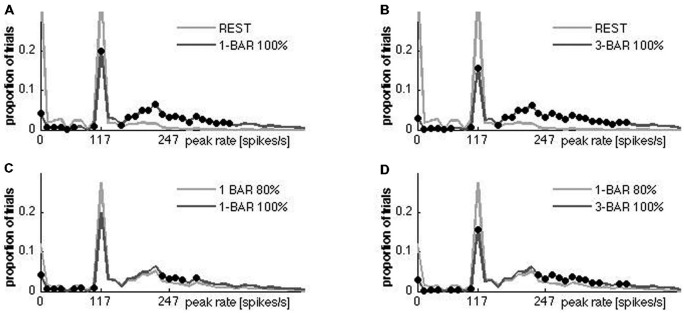
**Statistic comparison of multiunit peak spiking.** Histograms with 15 ms bins. For each bin, a one-way ANOVA with five factors (rest 1st and rest 2nd experiment, 1-bar-100% contrast, three-bars-100% contrast, and 1-bar_80% contrast) was performed (α = 0.05). A black dot indicates that the distributions were different at the corresponding point at the abscissa. In all stimulus conditions the number of trials with zero rate decreased, and the spiking increased. **(A,B)** 2500 trials per condition (experimental group). **(C)** All trials with 1-bar stimulus with 80% contrast (replication group) compared with all trials with 1-bar of 100% contrast. **(D)** All trials 1-bar 80% contrast (replication group) compared with all trials of the 3-bar stimulus with 100% contrast.

The distribution of peak rates for all trials of a neuron had two narrow peaks and a broad peak (Figures 7, 8). The first (narrow) peak, which is very small in the stimulus trials, corresponded to trials with no spikes in the time-interval; the second (narrow) peak, appeared at peak-rates ~120 spikes s^−1^, the third (broad) peak, which dominated the stimulus trials and was quite small in the rest trials, corresponded to rates >140 Hz. Only the number of trials changed below and above the 140 spikes s^−1^ threshold when the ferrets were stimulated with the bar, but the basic shape of the distribution remained (Figure 8). All neurons had trials with spiking at rest and with most trials spiking maximally at 120 spikes s^−1^. The peak rate distributions were reproducible at different bin sizes. These findings are consistent with a dynamical system containing a stable fixed-point attractor with a separatrix in which natural fluctuations vs. stimulation have a small vs. large probability to get the system across the separatrix.

**Figure 9:**
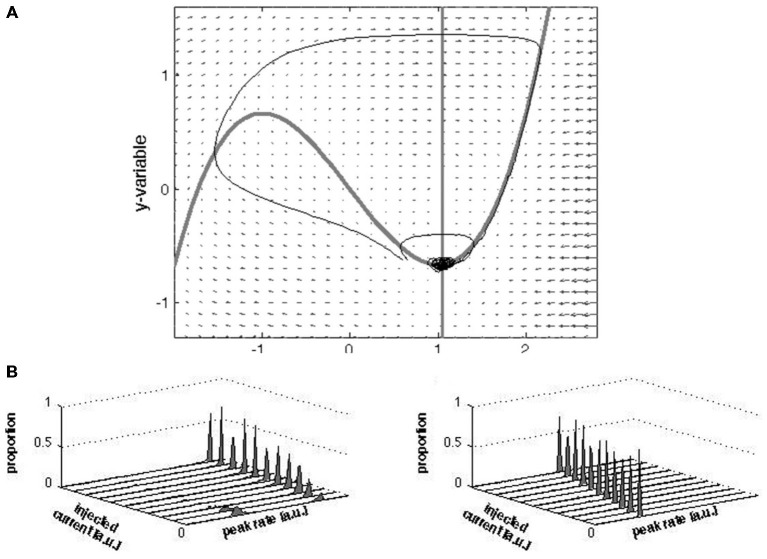
**Vector field, nullclines, trajectories and peak rate distributions of the modified FHN model. (A)** The small arrows represent the vectors; the nullclines are shown as the thick gray lines; the trajectories as (thin) black lines. Note that the two trajectories staring nearby (about [0.6, −0.6] diverge: one traverses a large “loop” through phase space and then converges towards the fixed point whereas the other immediately converges towards the fixed point). From this visualization it can be appreciated that the separatrix set apart distinct states. **(B)** Results of simulations of the peak firing rates with and without a separatrix. Increasing contrast was mimicked by increasing amplitudes of injected current (see “Materials and Methods” Section). The distributions were plotted as total number of trials normalized to one for comparison with (Figures 8A–D). In the presence of a separatrix, with increasing stimulation strength, the proportion of low peak firing rates vanishes while the proportion of high peak firing rates increase. The rate of the high peak firing slightly increases with increasing stimulation strength. In the absence of a separatrix the peak-firing rate simply scales with the stimulation strength.

We made a simple model that is topologically equivalent with our data only when it has a separatrix producing bi-modal peak rate distributions. The model is a modified Fitz-Hugh Nagumo model (see “Materials and Methods” Section). Without separatrix the model produces a uni-modal distribution of peak rates scaling with stimulus strength (Figure 9). With a separatrix, the bi-modality is recovered to a degree that depends (next to variation in the parameters *a*, *b* and *τ*) on the strength of the (model) stimulation: the stronger the stimulation, the larger the proportion of simulations in which the separatrix is crossed. This result is in agreement with the differences in peak rate distributions in Figure 8, in that stronger stimulation (1-bar 100% vs. 3-bar 100% and 1-bar 80% vs. 1-bar and 3-bar 100%) increased number of trials with peak rates >140 Hz.

**Figure 10:**
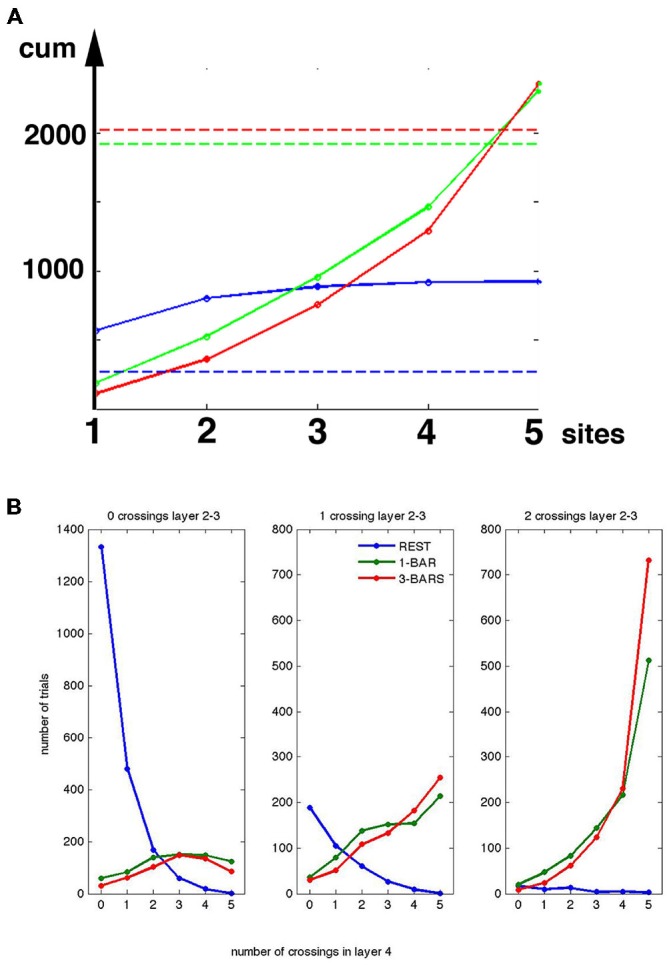
**Peak spike rates and crossing of the separatrix. (A)** *Y*-axis, cumulative number of trials with peak rates >140 Hz. *X*-axis 1, 2, 3, 4 and 5 leads (sites) in layer 4. For example, for *x* = 3, *y* = the number of trials with peak rates >140 Hz at lead 1 + lead 2 + lead 3. Rest trials (blue), 1-bar trials (green), and 3-bar trials (red). Stippled lines: total number of trials crossing the separatrix (total number of evoked trials), at rest (blue), 20 ms after stimulation and onwards for 1-bar (green); and 3-bar (red). **(B)** The number of trials in layers 2–3 (vertical axis) with peak spike rates <140 Hz (0 crossings), and number of trials with peak spiking rates >140 Hz (1 crossing and 2 crossings). *X*-axes, numbers of leads with spiking >140 Hz by the same trials in layer 4. **(A,B)** valid for the interval from 30–50 ms after stimulus onset.

The results in Figures 7–9 all indicate that the presence of a separatrix in the network of neurons may produce bi- or tri-modal peak rate distributions. The consistent minimum at 140 Hz could be taken as an effect of the separatrix separating the peak spike rates into trials with less and more than 140 Hz. Knowing the peak rate of each trial (Figure 7) and knowing whether that trial crossed the separatrix in the early post stimulus period (Figure 5A), we further tested whether crossings of the separatrix just after the start of the stimuli lead to peak rates above 140 Hz. A total of 1916 trials (out of 2500) crossed the separatrix in the 1-bar stimulus condition and 2021 trials in the 3-bar condition. Taking 140 Hz peak rate as an indication of the effect of the separatrix, the peak rate of a single trial might exceed this limit at 0, 1, 2, 3, 4 or 5 leads simultaneously. A total of 2309 trials in the 1-bar condition and 2356 trials in the 3-bar condition had peak-rates above 140 Hz (Figure 10). As we checked peak rate and separatrix crossing trial by trial, it turned out that the 2309 trials and the 2356 trials included all 1916 and 2021 trials crossing the separatrix. In other words, all trials crossing the separatrix had peak rates >140 Hz at 1, 2, 3, 4 or 5 leads. Thus, for all trials crossing the separatrix the peak rates increased above 140 Hz in one or more leads.

### The Separatrix Tends to Prevent Spontaneous Spiking from Propagating Through Layers

The layer 4 separatrix separates the spontaneous spiking from visually evoked spiking. One could therefore expect the separatrix to have a role in preventing spontaneous ongoing spiking from propagating through the cortical layers and perhaps propagating between cortical areas. Layer 2–3 multi-units also had a separatrix and a distribution of peak-rates with a minimum at 140 Hz. Assuming that the threshold corresponding to the separatrix for the layers 2–3 multiunit activity was at an instantaneous rate of 140 spikes s^−1^, and thus equal to that of the layer 4 neurons, we found the number of trials in which the 140 Hz threshold was crossed 0, 1, or 2 times in layers 2–3 (two leads) as a function of the number of crossings in layer 4 (five leads; see Figure 10). By far the majority of the stimulus trials crossing the 140 Hz threshold at more than two leads in layer 4 also crossed the 140 Hz threshold in layers 2–3. A total of 127 trials crossed the 140 Hz threshold at more than two sites in layer 4 during the rest condition. Of these less than 10, or 0.4% of the rest trials, crossed the 140 Hz threshold at two sites in layers 2–3 (Figure 10). Of the 1916 trials crossing the layer 4 separatrix in the 1-bar condition, 1348 trials also crossed the layers 2–3 separatrix and of the 2021 trials crossing the layer 4 separatrix in the 3-bar condition 1471 trials crossed the separatrix in the layers 2–3. Of the 277 rest trials crossing the layer 4 separatrix 52 trials crossed the separatrix in layers 2–3.

These results thus show that the separatrix in layer 4 plays a role in restricting weak and spontaneous transient increases in the number of spikes to influence the spiking in layers 2–3. As the axons from layer 4 neurons to a large extent end in layers 2–3 (Rockland, 1985), this suggests that the separatrices in layers 4 and 2–3 prevent the rest spiking dynamics from driving neurons to evoked dynamics in layers 2–3, whose neurons send axons to higher order visual areas (Rockland, 1985).

## Discussion

We presented evidence that the spontaneous ongoing spiking can dynamically be represented as a stable fixed-point attractor, and that the spontaneous spiking is limited to a bounded domain of the state space. Visually evoked spiking evolved in a separate, adjacent domain of state space that was locally set apart from the spontaneous state (domain) through a separatrix. In all evoked trials, the spiking returned to the spontaneous ongoing spiking domain where it resumed its spontaneous type trajectories. Finally, the number of sites in layer 4 at which the separatrix was crossed nonlinearly scaled with the number of separatrix crossings in the output layers 2 and 3.

The dynamics of the spontaneous ongoing and visually evoked spiking was reproducible and surprisingly simple given the many theoretical options (Guckenheimer and Holmes, 1983; Korn and Faure, 2003; Fdez Galán et al., 2004; Rabinovich et al., 2009; Churchland et al., 2010; Rajan et al., 2010; Shenoy et al., 2013; Woodman and Jirsa, 2013). Note that this result could not have been arrived at through (traditional) analysis of spike rates and spike timing but required the representation of the spiking activity in the state space, and the reconstruction of the vector fields in that space. The fixed point persisted in the three intervals defined in Figure 1. In isolation, this finding does not rule out that the stable fixed point vanishes in the brief windows (42 and 22 ms) separating these intervals. However, in some trials stimulation failed to induce the transit from the spontaneous state (domain) to the evoked state (domain), which indicates that the fixed point was persistent and stable during the brief windows (see also Figure 5).

As the spontaneous spiking attractor was stable throughout the stimulation period, and as we found no other attractive domains, our results are incompatible with the hypothesis that the sensory spiking replaces the spontaneous dynamics. They are also incompatible with the idea that evoked spiking is merely a modulation of spontaneous spiking; after all, the spiking evolved in different parts of the state space that are set apart by a separatrix, and the trajectories in the spontaneous and evoked state differed qualitatively. In contrast, our results indicate that whereas the dynamics of the ferret visual cortex layer 4 network is unaltered by visual stimulation, the spontaneous and evoked spiking are different states in that they evolve in different parts of the state space. Note that if the underlying network spiking dynamics is mono-stable and the visual evoked spiking states and spontaneous states are different and separate, it is not surprising that experimental sensory evoked data have been interpreted as modulations of spontaneous spiking and as replacing spontaneous spiking (Abeles et al., 1995; Arieli et al., 1995; Tsodyks et al., 1999; Fdez Galán et al., 2004; Fiser et al., 2004; Mazor and Laurent, 2005; Jones et al., 2007; Luzcak et al., 2009, 2013; Niell and Stryker, 2010). Our results reconcile the opposing hypotheses on how spontaneous ongoing spiking and sensory spiking relate. The spontaneous spiking dynamics does not exclude the emergence of complex spiking patterns (Rapp et al., 1985; Celletti and Villa, 1996). Also in this respect, our results are compatible with experimental results (Llinás and Paré, 1991; Fiser et al., 2004; Luzcak et al., 2009, 2013; Ringach, 2009; Destexhe, 2011).

Our results do not exclude the existence of more evoked spiking domains. In this context, reports showing a probabilistic difference in the number of spikes between two (experimental) conditions or a difference in spike sequences do not imply that these differences constitute two different spiking domains nor imply different spiking dynamics. Our initial visually evoked states were dependent on visual transients, which occur when the visual scene shifts or a saccade is made to a new point in the visual field (Müller et al., 2001). Other types of stimulus presentations may theoretically give rise to other spiking states, but this is still an open question.

Our finding that the ferret visual cortex (layer 4) dynamically acts as an excitable mono-stable system is significant in several respects. This dynamical organization enables the network to react fast to external drives (Figures 2–5), ensures that it will always returns to its resting state (Figures 2, 5), and evades the need for the network to re-organize, for example by creating another attractor, to pass from the spontaneous spiking state to the evoked spiking state. In other words, it guarantees robustness and speed of operation. Moreover, as the spontaneous ongoing and evoked spiking evolved in separate domains of state space, the classification of spikes as “signal” and “noise” becomes superfluous. At the same time, it should be noted that as the mathematics revealing the spiking dynamics does not include assumptions about coding and information content of the spiking in spontaneous states and during the evoked states, it is unable to reveal if spike patterns carrying such codes exist.

Glancing at the firing rates (Figures 1B,C), one could describe our data in terms of a baseline firing rate during spontaneous activity, which temporarily increases following stimulation onset (and offset), and subsequently reduces back to the spontaneous baseline activity. This behavior can in principle be produced by different dynamical systems, for instance by the linear model. Indeed, firing rate analysis does not allow one to identify the dynamics of the system’s collective behavior. This motivated the analysis using principal components, the subsequent vector field reconstruction as well as the peak rate distributions, without which we would not have been able to arrive at our reported results. Our analysis allowed us to show (rather then presume) why the return to baseline occurs, namely because the stable fixed point persists.

Regardless, physiologically, Na^+^ channel conductance cascades can bring silent neurons to spike. This is usually described as the membrane potential passing a threshold, above which the neurons start to spike. The separatrix setting apart the spontaneous ongoing spiking from the visual evoked spiking is something quite different: it signifies a functional threshold within the spiking domain. Effectively, its existence (near to the fixed point attractor) causes some neurons to sometimes remain in the spontaneous state whereas, inversely, some layer 4 neurons will sometimes enter visually evoked states in the absence of stimulation.

Our results suggest, however, that the incidental crossing of the separatrix in the absence of stimulation is not consequential: propagation of layer 4 activity to layers 2–3, and most likely other visual areas, scaled nonlinearly with the number of sites at the separatrix was crossed in layer 4: evokes states in layers 2–3 occurred primarily when the more than half of the recorded sites in layer 4 crossed the separatrix (Figure 10). Thus, the separatrices undoubtedly hamper most spontaneous ongoing spiking to become evoked, even in the output layers 2–3 of area 17 sending axons to other cortical areas (Figures 3, 4, 7, 8, 10).

Our type of anesthesia may have diminished the spiking and increased the stability. Whether the anesthesia increases the rest fixed-point attraction or makes the sensory drive weaker or moves the location of the separatrix does not affect dynamics as long as there are trials of visually evoked spiking. It is also unlikely that the separatrix is due to the anesthesia, because several neurons had peak rates exceeding 180 spikes s^−1^ or more in all trials and all neurons had trials with rest spiking. Also, in awake animals, sensory stimuli are associated with peak fining rates that are not distinguishable from resting activity in a considerable number of trials (Niell and Stryker, 2010; Luzcak et al., 2013).

The spiking of all neurons and the spiking in single trials were examined, demonstrating consistent mono-stable dynamics, a separatrix, and two different spiking state domains. This temporal dynamics is a succinct mathematical description of the temporal interactions of the neurons in layers 4 and 2–3 at the network scale and such a description is difficult to obtain by other methods. This description makes it easier to study whether and to what extent experimental manipulations of membrane conductances and spiking is able (or not) to alter this dynamics. Our results were reproducible over scales ranging from single trials, single neurons, to multi-units, populations of neurons, animals, and different groups of animals. Thus the dynamics, given by the stable fixed point and the separatrix, and the spiking states show the mechanics of the collective behavior of neurons of the neuron network in area 17 mapping visual objects in the center of field of view.

## Author Contributions

PER designed the experiment. ZD and SV conducted the experiments. RH and PER analyzed the data. PER and RH wrote the manuscript. RH, VKJ, and PER discussed the results and made the theoretical interpretation. All authors contributed to editing the final version of the manuscript.

## Conflict of Interest Statement

The authors declare that the research was conducted in the absence of any commercial or financial relationships that could be construed as a potential conflict of interest.

## References

[B1] Abeles M. Bergman H. Gat I. Meilljson I. Seidemann E. Tishby N.. (1995). Cortical activity flips among quasi-stationary states. Proc. Natl. Acad. Sci. U S A 92, 8616–8620. 10.1073/pnas.92.19.8616 7567985 PMC41017

[B2] Arieli A. Shoham D. Hildesheim R. Grinvald A. (1995). Coherent spatiotemporal patterns of ongoing activity revealed by real-time optical imaging coupled with single unit recording in the cat visual cortex. J. Neurophysiol. 73, 2072–2093. 7623099 10.1152/jn.1995.73.5.2072

[B3] Averbeck B. B. Latham P. E. Pouget A. (2006). Neural correlations, population coding and computation. Nat. Rev. Neurosci. 7, 358–366. 10.1038/nrn1888 16760916

[B4] Benjamini Y. Hochberg Y. (1995). Controlling the false discovery rate: a practical and powerful approach to multiple testing. J. R. Stat. Soc. B Methodol. 57, 289–300.

[B5] Celletti A. Villa A. E. P. (1996). Low-dimensional chaotic attractors in the rat brain. Biol. Cybern. 74, 387–393. 10.1007/s004220050250 8991454

[B6] Churchland M. M. Yu B. M. Cunningham J. P. Sugrue L. P. Cohen M. R. Corrado G. S.. (2010). Stimulus onset quenches neural variability: a widespread cortical phenomenon. Nat. Neurosci. 13, 369–378. 10.1038/nn.2501 20173745 PMC2828350

[B7] Curto C. Sakata S. Marguet S. Itskov V. Harris K. D. (2009). A simple model of cortical dynamics explains variability and state dependence of sensory responses in urethane-anesthetized auditory cortex. J. Neurosci. 29, 10600–10612. 10.1523/JNEUROSCI.2053-09.2009 19710313 PMC2861166

[B8] Daffertshofer A. (2010). “Benefits and pitfalls in analyzing noise in dynamical systems—On stochastic differential equations and system identification,” in Nonlinear Dynamics in Human Behavior, Studies in Computational Intelligence (Vol. 328), eds Huys R. Jirsa V. K. (Berlin: Springer-Verlag), 35–68.

[B9] Daffertshofer A. Lamoth C. J. Meijer O. G. Beek P. J. (2004). PCA in studying coordination and variability: a tutorial. Clin. Biomech. (Bristol, Avon) 19, 415–428. 10.1016/j.clinbiomech.2004.01.005 15109763

[B10] Destexhe A. (2011). Intracellular and computational evidence for a dominant role of internal network activity in cortical computations. Curr. Opin. Neurobiol. 21, 717–725. 10.1016/j.conb.2011.06.002 21715156

[B11] Fdez Galán R. Sachse S. Galizia C. G. Herz A. V. M. (2004). Odor-driven attractor dynamics in the antennal lobe allow for simple and rapid olfactory pattern classification. Neural Comput. 16, 999–1012. 10.1162/089976604773135078 15070507

[B12] Fiser J. Chiu C. Weliky M. (2004). Small modulation of ongoing cortical dynamics by sensory input during natural vision. Nature 43, 573–578. 10.1038/nature02907 15457262

[B13] Fitz-Hugh R. A. (1961). Impulses and physiological states in theoretical models of nerve membrane. Biophys. J. 1, 445–466. 10.1016/s0006-3495(61)86902-6 19431309 PMC1366333

[B14] Guckenheimer J. Holmes D. (1983). Non-Linear Oscillations, Dynamical Systems and Bifurcations of Vector Fields. New York: Springer Verlag.

[B16] Harvey M. A. Valentiniene S. Roland P. E. (2009). Cortical membrane potential dynamics and laminar firing during object motion. Front. Syst. Neurosci. 3:7. 10.3389/neuro.06.007.2009 19753323 PMC2742661

[B18] Hubel D. H. Wiesel T. N. (1959). Receptive fields of single neurons in the cat’s striate cortex. J. Physiol. 148, 574–591. 10.1113/jphysiol.1959.sp006308 14403679 PMC1363130

[B180] Innocenti G. M. Manger P. R. Masiello I. Colin L. Tettoni L. (2002). Architecture and callosal connections of visual areas 17, 18, 19, and 21 in the ferret *(Mustela putorius)*. Cereb. Cortex 12, 411–422. 10.1093/cercor/12.4.411 11884356

[B19] Jones L. M. Fontanini A. Sadacca B. F. Miller P. Katz D. B. (2007). Neural stimuli evoke dynamic sequences of states in sensory ensembles. Proc. Natl. Acad. Sci. U S A 104, 18772–18777. 10.1073/pnas.0705546104 18000059 PMC2141852

[B20] Jung R. (1959). “Microphysiologie corticaler neurone: ein beitrag zur koordination der hirnrinde und des visuellen systems,” in Structure and Function of the Cerebral Cortex, eds Tower D. B. Schadé J. P. (Amsterdam: Elsevier), 204–233.

[B22] Korn H. Faure P. (2003). Is there chaos in the brain II. Experimental evidence and related models. C. R. Biol. 326, 787–840. 10.1016/j.crvi.2003.09.011 14694754

[B23] Llinás R. R. Paré D. (1991). Of dreaming and wakefulness. Neuroscience 44, 521–535. 10.1016/0306-4522(91)90075-y 1754050

[B24] Luzcak A. Barthó P. Harris K. D. (2009). Spontaneous events outline the realm of possible sensory responses in neocortical populations. Neuron 62, 413–425. 10.1016/j.neuron.2009.03.014 19447096 PMC2696272

[B25] Luzcak A. Bartho P. Harris K. D. (2013). Gating of sensory input by spontaneous cortical activity. J. Neurosci. 33, 1684–1695. 10.1523/JNEUROSCI.2928-12.2013 23345241 PMC3672963

[B26] Mazor O. Laurent G. (2005). Transient dynamics versus fixed points in odor representations by locust antennal lobe projection neurons. Neuron 48, 661–673. 10.1016/j.neuron.2005.09.032 16301181

[B28] Müller J. R. Mehta A. B. Krauskopf J. Lennie P. (2001). Information conveyed by onset transients in responses of striate cortical neurons. J. Neurosci. 21, 6978–6990. 11517285 10.1523/JNEUROSCI.21-17-06978.2001 PMC6763098

[B29] Nagumo J. Arimoto S. Yoshizawa S. (1962). An active pulse transmission line simulating nerve axon. Proc. IRE 50, 2061–2070. 10.1109/jrproc.1962.288235

[B30] Niell C. M. Stryker M. P. (2010). Modulation of visual responses by behavioral state in mouse visual cortex. Neuron 65, 472–479. 10.1016/j.neuron.2010.01.033 20188652 PMC3184003

[B31] Quiroga R. Q. Nadasdy Z. Ben-Shaul Y. (2004). Unsupervised spike detection and sorting with wavelets and superparamagnetic clustering. Neural Comput. 16, 1661–1687. 10.1162/089976604774201631 15228749

[B32] Rabinovich M. Huerta R. Laurent G. (2009). Transient dynamics for neural processing. Science 321, 48–50. 10.1126/science.1155564 18599763

[B33] Rajan K. Abbott L. F. Sompolinsky H. (2010). Stimulus-dependent suppression of chaos in recurrent neural networks. Phys. Rev. E Stat. Nonlin. Soft Matter Phys. 82:011903. 10.1103/physreve.82.011903 20866644 PMC10683875

[B34] Rapp P. E. Zimmerman I. D. Albano A. M. Deguzman G. C. Greenbaun N. N. (1985). Dynamics of spontaneous neural activity in the simian motor cortex: the dimension of chaotic neurons. Phys. Lett. 110A, 335–338. 10.1016/0375-9601(85)90786-8

[B35] Rieke F. Warland D. van Steveninck de R. Bialek W. (1999). Spikes: Exploring the Neural Code. Cambridge, MA: The MIT Press.

[B36] Ringach D. L. (2009). Spontaneous and driven cortical activity: implications for computation. Curr. Opin. Neurobiol. 19, 439–444. 10.1016/j.conb.2009.07.005 19647992 PMC3319344

[B37] Rockland K. (1985). Anatomical organization of primary visual cortex (area 17) of the ferret. J. Comp. Neurol 241, 225–236. 10.1002/cne.902410209 4067016

[B38] Roland P. E. (2010). Six principles of visual cortical dynamics. Front. Syst. Neurosci. 4:28. 10.3389/fnsys.2010.00028 20661451 PMC2906257

[B380] Shenoy K. V. Sahani M. Churchland M. M. (2013). Cortical control of arm movements: a dynamical systems perspective. Ann. Rev. Neurosci. 36, 337–359. 10.1146/annurev-neuro-062111-150509 23725001

[B40] Tsodyks M. Kenet T. Grinvald A. Arieli A. (1999). Linking spontaneous activity of single cortical neurons and the underlying functional architecture. Science 286, 1943–1946. 10.1126/science.286.5446.1943 10583955

[B42] Woodman M. Jirsa V. K. (2013). Emergent dynamics from spiking neuron networks through symmetry breaking of connectivity. PLoS One 8:64339. 10.1371/journal.pone.0064339 23691200 PMC3656844

